# TIME-USE AND WELL-BEING IN FAMILY AND OTHER UNPAID CAREGIVERS OF OLDER ADULTS

**DOI:** 10.1093/geroni/igac059.1406

**Published:** 2022-12-20

**Authors:** Sol Baik, Amanda Lehning, Paul Sacco, John Cagle, Nancy Kusmaul

**Affiliations:** University of Virginia Weldon Cooper Center for Public Service, Charlottesville, Virginia, United States; University of Maryland, Baltimore, Baltimore, Maryland, United States; University of Maryland, Baltimore, Baltimore, Maryland, United States; University of Maryland, Baltimore, Baltimore, Maryland, United States; University of Maryland Baltimore County, Baltimore, Maryland, United States

## Abstract

Due to the intensive time commitment for caregiving, caregivers report limited freedom to engage with others, participate in physical activities, pursue leisure activities, and have adequate time for sleep. Few studies have focused on caregivers’ time-use across different activities, particularly how different patterns of time-use are associated with well-being. This study aimed to: (1) identify time-use profiles of family caregivers of older adults and (2) examine associations between identified time-use profiles and caregiver well-being. We analyzed 1,640 family caregivers of community-dwelling older adults by combining secondary data from Round 7 (2017) of the National Study of Caregiving and the National Health and Aging Trends Study. We conducted latent profile analysis to estimate time-use profiles including covariates and outcomes. Three classes of caregivers emerged based on time-use patterns. The High Committed class (20%) spent the longest time in non-eldercare related committed activities, such as household activities and paid work. The High Discretionary class (49%) spent the highest amount of discretionary time, including social activities, physical activities, and other free-time activities. They also spent the least amount of non-eldercare committed time compared to the other two caregiver types. Lastly, the Balanced class (31%) allocated time relatively evenly in all activities. When comparing well-being outcomes between time-use profiles, caregivers in the High Discretionary class had worse self-rated health but lower levels of anxiety than the Balanced class. Research on time-use and caregiver well-being may help identify at-risk caregiver groups based on lifestyle profiles and develop targeted policies to promote better caregiver well-being.

